# Pediatric acute hospitalization for anorexia nervosa: an economic evaluation

**DOI:** 10.1186/s13052-024-01605-0

**Published:** 2024-02-27

**Authors:** Maria Rosaria Marchili, Elena Bozzola, Stefano Guolo, Silvio Marchesani, Giulia Spina, Cristina Mascolo, Stefano Vicari, Isabella Tarissi De Jacobis, Massimiliano Raponi, Alberto Villani

**Affiliations:** 1 Pediatric Unit, Bambino Gesù Children’s Hospital, IRCCS, Rome, Italy; 2 Sanitary Direction, Bambino Gesù Children’s Hospital, IRCCS, Rome, Italy; 3 Child Neuropsychiatry Unit, Bambino Gesù Children’s Hospital, IRCCS, Rome, Italy

**Keywords:** Anorexia nervosa, Hospitalization, Hospitalization acute cost, Children, Covid-19

## Abstract

**Background:**

Anorexia nervosa (AN) is a psychiatric disorders which may potentially led to a high risk of health medical complications, suicide and self-harming behaviour. Since Covid-19 pandemic onset in March 2020, evidence suggested an increase occurrence of AN. The main aim of the retrospective analysis is to define the cost of hospitalization in the acute phase (HAP) at IRCCS Bambino Gesù Children Hospital, Rome, Italy, over 2 years study. Secondary purposes are defining the main risk factors for a prolonged hospitalization (including age, sex and comorbidities) and the possible influence of Covid-19 pandemic on AN admission and hospital stay.

**Methods:**

for the purpose of the study, we included children and adolescents aged less than 18 years, admitted to IRCCS Bambino Gesù Children Hospital, Rome, Italy, with a diagnosis of AN. Medical costs were calculated consulting the Lazio Regional Health Service Tariffs. Basing on the date of hospital admission, patients were later divided into two subgroups: subgroup A included patients hospitalized prior than Covid-19 onset (from March 2019 to February 2020) and subgroup B those admitted after (from March 2020 to October 2022).

**Results:**

a total of 260 patients has been included in the study with a median age of 15 years (range 6–18 years). The total health care cost of AN hospitalized patients was of EUR 3,352,333 with a median cost of EUR 11,124 for each admission (range EUR 930 − 45,739) and a median daily cost of EUR 593 (range EUR 557–930). Median cost was higher in case of comorbidities, guarded patients, enteral feeding. A prolonged hospitalization has been documented in subgroup A with a higher economic burden.

**Conclusions:**

the economic burden of eating disorders is of note. Adequate sanitary policies as well as health economic analyses are required to gain insight into the cost-effectiveness of AN management.

**Trial registration:**

2526-OPBG-2021.

## Background

Anorexia nervosa (AN) may be defined as a psychiatric disorder that ravages both mind and body, characterized by restriction of food intake leading to starvation, malnutrition and risk of dying for health complications, suicide or self-harming attitudes. In minors, a precise cutoff regarding low body mass index (BMI) to define AN has not been established, since many factors should be considered, including age, sex, BMI before the start of symptoms and the rapidity of weight loss. Intense fear of weight gain is a central feature as well as an obsessive focus on weight and body image, combined with a willing of complete control over everything that is eaten, including the preparation of food [1]. The major cause for the medical complications in AN is the imbalance between energy intake and requirements, leading to a hypometabolic state. Medical complications are related to weight loss and malnutrition and may include cardiovascular abnormalities, hypotension, hypokalemia, osteoporosis, reduced growth velocity and neurocognitive alterations. Detailed medical, psychiatric, nutritional assessment as well as physical examination and laboratory testing are required to identify complications and comorbidities. In life-threatening cases, an acute phase hospitalization is immediately required to prevent further deterioration of clinical conditions or to contain suicide risk. Psychosocial, psychotherapeutic and pharmacological treatments are associated to nutritional rehabilitation.

Since Covid-19 pandemic onset in March 2020, evidence suggested an increase occurrence of AN, mostly among adolescents, likely correlated to stress, isolation and depression. By the way, an Italian multicentric study revealed that, despite a 48.2% decline of admissions at Emergency Departments, there was a significant increase (83.1%) inpatient admissions for neuropsychiatric problems, including suicidal ideation (+ 147%), depression (+ 115%), eating disorder (+ 78.4%). During the pandemic period, a 39.5% increase in neuropsychiatric disorders (NPD) hospitalizations was observed as well, suggesting that Covid-19 pandemic had a major impact on children’s health, mainly on their NPD development [2]. 

The main aim of the retrospective analysis is to define the cost of hospitalization in the acute phase (HAP) at Bambino Gesù Children Hospital, Rome, Italy, over 2 years study. Secondary purposes are defining the main risk factors for a prolonged hospitalization (including age, sex and comorbidities) and the possible influence of Covid-19 pandemic on AN admission and hospital stay.

## Methods

For the purpose of the study, we included children and adolescents aged less than 18 years, admitted to IRCCS Bambino Gesù Children Hospital, Rome, Italy, with a diagnosis of AN. The period study ranged from 1st March 2019 until 31st October 2022. AN has been defined according to literature and to the diagnostic criteria in the *Diagnostic and Statistical Manual of Mental Disorders* of the American Psychiatric Association, fifth edition [3]..

Patients were excluded if they did not meet the inclusion criteria. As for the enrolled patients, medical costs were calculated consulting the Lazio Regional Health Service Tariffs. Procedure codes were used, in order to precisely calculate the cost of any single exam and therapy. For any patient, the HAP has been calculated. The final value includes cost of hospital accommodation and management at the General Pediatric Disease Unit. To this cost, the price of procedures (imaging, laboratory exams, medical and paramedical evaluations) and medical treatments was added. The cost analysis was performed using an Excel database reporting the cost for each patient correlated to laboratory and imaging exams, specialist evaluations (for example, nutritionist or psychiatric consultant), therapy, and hospital accommodation.

Basing on the date of hospital admission, patients were later divided into two subgroups: subgroup A included patients hospitalized prior than Covid-19 onset (from March 2019 to February 2020) and subgroup B those admitted after (from March 2020 to October 2022).

In order to compare and correlate data, a statistical analysis was performed. The comparison study among subgroups was performed using the Student t-test (two-sided) for parametric distribution or Mann-Whitney test for nonparametric distribution. Moreover, Chi-squared test or Fisher’s exact test (when appropriate) were performed to compare proportions or categorical outcomes. The regression analysis was studied using Spearman test. Data with statistical significance had a *p*-value less than 0.05. Statistical analysis was performed using the GraphPad Prism software, version 5 for Machintosh (GraphPad Software, Inc).

## Results

The present study investigated the economic burden of hospitalized patients affected by AN between 1st May 2020 till 1st May 2022 at Bambino Gesù Children Hospital, in addition to evaluating pre-Covid-19 and post- Covid19 (subgroups A and B) influence on AN prevalence and costs. A total of 260 patients has been included in the study (17 males vs. 243 females), median age 15 years (range 6–18 years). Other neuropsychological comorbidities, including suicide attempt, psychotic or anxious disorders, have been found in a percentage of 24.2% (*n* = 63) with a total of 4.2% of guarded patients (*n* = 11). A percentage of 43.1% (*n* = 112) required enteral feeding (EF); a median duration of 3 days has been detected between the admission and EF onset (range 0–23 days). General data are summarized in Tables 1 and 2.

**Table 1:** General characteristics of the study cohort. Subgroup A: from March 2019 to March 2020. Subgroup B: from March 2020 to October 2022

Parameters	Value
Number of patients	260
Median age in years (range)	15 (6–18)
Number of males/females (%)	17/243 (6.5/93.5)
Median length of hospitalization in days (range)	19 (1–65)
Number of patients hospitalized in subgroup A (%)	36 (13.8)
Number of patients hospitalized in subgroup B (%)	224 (86.2)
Number of guarded patients (%)	11 (4.2)
Number of patients who required enteral feeding (%)	112 (43.1)
Median days between admission and the start of enteral feeding (range)	3 (0–23)
Number of patients who presented comorbidities (%)	63 (24.2)
Number of patients with mood disorder (%)	39 (15)
Number of patients with anxious spectrum disorder (%)	14 (5.4)
Number of patients with obsessive compulsive disorder (%)	8 (3.1)
Number of patients with other comorbidities (%)	16 (6.1)
Total health care cost for hospitalizations in EUR	3,352,333
Median cost for each hospitalization in EUR (range)	11,124 (930-45739)
Median daily cost in EUR (range)	593 (557–930)

**Table 2:** Therapies and exams performed during hospitalizations. CBC: complete blood count. Chem: chemistry panel. EKG: electrocardiogram. MRI: magnetic resonance image

Therapies	
*Parameters. Number of patients who underwent*	*Value*
fluid therapy (%)	252 (96.9)
supplements therapy (%)	233 (89.6)
therapy with aripiprazole (%)	209 (80.4)
therapy with sertraline (%)	136 (52.3)
therapy with fluoxetine (%)	27 (10.4)
therapy with olanzapine (%)	16 (6.1)
therapy with alprazolam (%)	14 (5.4)
therapy with risperidone (%)	4 (1.5)
Blood exams	
*Parameters. Number of patients who underwent*	*Value*
CBC and Chem analysis (%)	*256 (98.5)*
vitamin dosages (%)	221 (85)
screening for celiac disease (%)	208 (80)
hormonal dosages (%)	170 (65.4)
urine exams (%)	124 (47.7)
at least 1 blood gas analysis (%)	22 (8.5)
Other exams	
*Parameters. Number of patients who underwent*	*Value*
at least 1 EKG (%)	241 (92.7)
abdomen ultrasound (%)	202 (77.7)
cerebral MRI (%)	99 (38.1)
echocardiogram (%)	71 (27.3)
Holter EKG (%)	19 (7.3)
peripheral vessels doppler ultrasound (%)	16 (6.1)

The total health care cost of AN hospitalized patients was EUR 3,352,333 with a median cost of EUR 11,124 for each admission (range EUR 930 − 45,739) and a median daily cost of EUR 593 (range EUR 557–930).

Considering patients age (cut-off of 14 years), no statistically significant differences have been detected as for length of hospitalization (LOS) and economic related burden between males and females. The comparisons between these specific subgroups are shown in Table 3. (Table 3)

Median LOS was statistically significant higher in patients with comorbidities than patients without comorbidities (23 days, range 3–65 days vs. 17 days, range 1–55 days; *p*-value 0.006). Similarly, median cost of AN patient with comorbidities was significantly greater than AN patient without comorbidities (EUR 13,485, range EUR 1814-37,959 vs. EUR 10,323, range EUR 930 − 45,739; *p*-value 0.005).

Patients may need a supervision of activity at all times, including meals and snacks and bathroom privileges by a 1:1 monitoring. These guarded patients had a higher prevalence of comorbidities compared to not guarded ones (91% vs. 23.3%; *p*-value 0.00001). Those patients required more frequently EF (81.8% vs. 41.4%; *p*-value 0.008) with a longer median LOS compared to not-guarded patients (41 days, range 25–55 days vs. 18 days, range 1–65 days) with a higher median medical expenditure (EUR 33,015, range 15,776 − 45,739 vs. EUR 10,703, range 930 − 37,673; *p*-value 0.00001).

Median LOS of patients treated with EF was significantly higher compared to median LOS of patients without EF (28 days, range 9–65 days vs. 14 days, range 1–47 days; *p*-value 0.00001) with a greater median hospitalization total cost (EUR 16,391, range EUR 5306-45,739 vs. EUR 8580, range EUR 930 − 43,143; *p*-value 0.00001).

The comparisons between these specific subgroups are shown in Table 3. (Table 3)

**Table 3:** Comparison between gender, age, comorbidities, needing of guarding, needing of enteral feeding subgroups. *Results with statistical significance

Gender			
*Value*	*Females*	*Males*	*p-value*
Median age in years (range)	15 (6–18)	14 (8–17)	0.045*
Median length of hospitalization in days (range)	19 (1–65)	15 (5–47)	0.098
Median cost of hospitalization in EUR (range)	11,373 (930 − 45,739)	8703 (3072-43,143)	0.97
Number of patients with comorbidities (%)	58 (23.8)	5 (29.4)	0.61
Number of guarded patients (%)	10 (4.3)	1 (6.2)	0.53
Number of patients who required enteral feeding (%)	107 (44)	3 (21.4)	0.041*
Age			
*Value*	*< 14 years*	*≥ 14 years*	*p-value*
Median length of hospitalization in days (range)	19 (4–57)	19 (1–65)	0.726
Median cost of hospitalization in EUR (range)	11,509 (2584-36,136)	10,900 (930 − 45,739)	0.098
Median days between admission and the start of enteral feeding (range)	4 (0–14)	3 (0–23)	0.529
Comorbidities			
*Value*	*With comorbidities*	*Without comorbidities*	*p-value*
Median age in years (range)	15 (8–18)	15 (6–18)	0.342
Median length of hospitalization in days (range)	23 (3–65)	17 (1–55)	0.006*
Median cost of hospitalization in EUR (range)	13,485 (1814-37,959)	10,323 (930 − 45,739)	0.005*
Median days between admission and the start of enteral feeding (range)	3 (0–23)	4 (1–9)	0.741
Number of guarded patients (%)	10 (91)	53 (23.3)	0.00001*
Number of patients who required enteral feeding (%)	35 (23.8)	28 (24.7)	0.765
Guarded patients			
*Value*	*Guarded patients*	*Non-guarded patients*	*p-value*
Median age in years (range)	15 (6–18)	15 (12–17)	0.896
Median length of hospitalization in days (range)	41 (25–55)	18 (1–65)	0.00001*
Median cost of hospitalization in EUR (range)	33,015 (15,776 − 45,739)	10,703 (930 − 37,673)	0.00001*
Median days between admission and the start of enteral feeding (range)	3 (0–23)	4 (1–9)	0.912
Number of patients who required enteral feeding (%)	9 (81.8)	103 (41.4)	0.008*
Enteral feeding			
*Value*	*EF patients*	*Non-EF patients*	*p-value*
Median age in years (range)	15 (6–18)	15 (9–18)	0.726
Median length of hospitalization in days (range)	14 (1–47)	28 (9–65)	0.00001*
Median cost of hospitalization in EUR (range)	16,391 (5306-45,739)	8580 (930 − 43,143)	0.00001*

Concerning differences between subgroup A and B, a greater median LOS has been documented in subgroup A (29 days, range 5–65 days vs. 18 days, range 1–57 days; *p*-value 0.0008) with a higher economic burden considering hospitalization median cost (EUR 16,758, range EUR 3072-45,739 vs. EUR 10,695, range EUR 930 − 37,959; *p*-value 0.0016). A statistically significant difference has been found considering median timing between the AN patient’s admission and EF onset comparing pre and post-Covid19 pandemic (8 days, range 1–14 days vs. 3 days, range 0–23 days; *p*-value 0.0026). Conversely, no statistically significant differences have been described between the two subgroups comparing sex, comorbidities, EF needing (Table 4).

**Table 4:** Main differences between patients hospitalized prior than Covid-19 onset (from March 2019 to February 2020, Subgroup A), and patients hospitalized after Covid-19 onset (from March 2020 to October 2022, Subgroup B). *Results with statistical significance

Value	Subgroup A	Subgroup B	*p*-value
Number of male patients (%)	5 (13.9)	12 (5.4)	0.0681
Number of female patients (%)	31 (86.1)	212 (94.6)	0.0681
Median age in years (range)	14 (9–17)	15 (6–18)	0.014*
Median length of hospitalization in days (range)	29 (5–65)	18 (1–57)	0.0008*
Median days between admission and the start of enteral feeding (range)	8 (1–14)	3 (0–23)	0.0026*
Median cost of hospitalization in EUR (range)	16,758 (3072-45,739)	10,695 (930 − 37,959)	0.0016*
Number of patients with comorbidities (%)	11 (30.5)	52 (30.2)	0.911
Number of guarded patients (%)	7 (19.4)	4 (1.8)	0.0001*
Number of patients who required enteral feeding (%)	12 (33.3)	100 (44.6)	0.21

Moreover, a significant positive correlation has been detected between admission, latency of EF onset and total hospitalization costs (Rs 0.2, *p*-value 0.047).

## Discussion

The main finding of our study is the high acute hospitalization cost (AHC) associated with AN in pediatric patients. Three key factors, namely comorbidities, EF and guarding, correlated to a prolonged LOS and to a higher AHC.

Hospitalization is required mainly in malnourished cases. Indications for hospitalization include starvation with profound hypotension or dehydration, severe electrolyte abnormalities, arrhythmias or severe bradycardia, suicide risk [1]. One of the most significant medical complications, which often leads to hospitalization, is vital sign instability, including bradycardia and orthostatic hypotension, caused by the underlying malnutrition and increased vagal tone. Electrolyte imbalances as well may occur in patients who engage in purging behaviors or in excessive water drinking. For children and pre-adolescents, the goal is to correct undernutrition, to return to the individual’s growth curve for proper growth and pubertal development. Evidence exists that substantial weight gain is best achieved in inpatient settings, requiring a multi-specialist approach [4]. Most cases may require a prolonged LOS due to the latency of initial EF strategies approach [5]. At first, nutritional therapy includes supervised meals and additional liquid supplements to prevent dehydration. Nevertheless, in severely emaciated patients at high medical risk, NE, under professional and supportive supervision, should be considered. In case of failure of the above nutritional strategies, parenteral nutrition is even indicated. The risk of a refeeding syndrome, which can lead to delirium, stupor, seizures, coma and death, especially during the early stages, should be kept in mind. So, a monitored program of nutrition, a strict control of parameters and periodic blood examination should be scheduled in order to prevent this fatal complication, especially in those with a severe malnutrition status.

The indication for close psychiatric monitoring of safety concerns, including suicidal thoughts, is also to be stressed. AN is often associated to comorbid disorders, mainly depression and anxiety disorders, which should always be screened for as they can further complicate treatment. Of note, depression and anxiety symptoms should be linked to AN. It may be challenging teasing apart primary depressive and anxiety disorders from depressive and anxiety symptoms caused by malnutrition. In fact, there is a consistent overlap in symptoms and signs [6]. Suicidal ideation and self-injurious behavior may complicate depression, anxiety and AN, requiring dedicated surveillance [7]. 

In our study, the average HAC for patient is EUR 11,124. The high economic burden is in line with literature, which, analyzing cost-effectiveness intervention report high economic cost over short and long period analysis [8, 9]. 

Finally, during the Covid-19 pandemic period, there was a raise in the number of admissions to the emergency departments of patients with AN, having a more severe general status, psychiatric comorbidities and dehydration [10]. 

At the same time, an increase of AN adolescent’s hospitalization rate has been observed. These findings should raise awareness of the mental difficulties experienced by children and adolescents during the Covid-19 pandemic. Our result are in line with the systematic revision of literature which reported on average a 48% increase in eating disorder admissions during the pandemic compared to previous timepoints [11]. In fact, after pandemic onset, the increased stress caused by the Covid-19 circulation and the collective sense of lack of personal control likely contributed to increase neuropsychological disorders in adolescents [2, 12, 13]. 

Nevertheless, in our study, the LOS of AN adolescents was reduced compared to pre-pandemic period. A possible explanation is the rapid start of EF during Covid-19 pandemic (3 days, range 0–23 days) which accelerated the discharge of patients. During the current pandemic, moreover, face-to-face clinical assessments, psychological and psychopathological treatments were reduced to minimize unnecessary physical contact and hence potential exposure to the virus [14]. So, both medical staff and families’ supporting discharge as soon as possible, which might have led to increased motivation to adhere to the AN treatment protocol and reduce psychopathological and psychological assessment, influenced the shorter duration of hospitalization.

No statistically significant differences have been described between pre and post pandemic among patients comparing sex, comorbidities, EF needing in our study. Our results are in contrast with Girardi M et al. who found out more intravenous fluids, oral dietetic supplements and enteral nutrition by nasogastric tube in post Covid-19 period than before the onset of pandemic. Moreover, the same group presented higher prevalence of psychiatric comorbidities and required most frequently treatments with psychotropic drugs [15]. Nevertheless, our result is in line with Goldberg et al. who reported a 35% reduction in median hospitalization duration even if there was a significant increase in the number of adolescents hospitalized with AN [16]. Different findings regarding eating disorder studies may correlate to the variability of restrictive public health measures and to the effects on the population.

## Conclusion

The economic burden of eating disorders is of note, although the available evidence probably under-estimates the costs. In fact, indirect costs, such as parents working missed days, have not been included in the cost-analysis study. As a conclusion, adequate sanitary policies as well as health economic analyses to gain insight into the cost-effectiveness of AN treatment are required.

## Data Availability

dataset is available at Marchili’s study room and data will be made available on reasonable request.

## Abbreviations

AN: Anorexia nervosa
BMI: Body mass index
Covid-19: Coronavirus disease of 2019
NPD: Neuropsychiatric disorders
HAP: Hospitalization in the acute phase
EF: Enteral feeding
LOS: Length of hospitalization
CBC: Complete blood count
Chem: Chemistry panel
EKG: Electrocardiogram
MRI: Magnetic resonance image
AHC: Acute hospitalization cost
